# HMGB1 as a Potential Biomarker and Therapeutic Target for Malignant Mesothelioma

**DOI:** 10.1155/2019/4183157

**Published:** 2019-02-12

**Authors:** Yanbin Wang, Zhaoqiang Jiang, Jianing Yan, Shibo Ying

**Affiliations:** ^1^Institute of Occupational Diseases, Zhejiang Academy of Medical Sciences, Hangzhou 310013, China; ^2^Department of General Surgery, Sir Run Run Shaw Hospital Affiliated to Medical College of Zhejiang University, Hangzhou 310016, China

## Abstract

Malignant mesothelioma (MM) is a rare, aggressive, and highly lethal cancer that is substantially induced by exposure to asbestos fibers. High-mobility group box 1 (HMGB1) is an intriguing proinflammatory molecule involved in MM. In this review, we describe the possible crucial roles of HMGB1 in carcinogenic mechanisms based on *in vivo* and *in vitro* experimental evidence and outline the clinical findings of epidemiological investigations regarding the possible roles of HMGB1 as a biomarker for MM. We conclude that novel strategies targeting HMGB1 may suppress MM cells and interfere with asbestos-induced inflammation.

## 1. Introduction

Malignant mesothelioma (MM), which originates from the mesothelial cells that form the lining of the viscera, is a rare, aggressive, and highly lethal cancer. Extensive epidemiological evidence to date shows that around the world, this cancer is predominantly induced by chronic exposure to natural asbestos and asbestiform fibers [1–3]. MM is mostly induced by occupational asbestos fibers. However, in some regions such as Turkey, MM is also due to environmental exposure to asbestos fibrous rock or erionite mines, materials that were used for building houses and paving roads [4, 5]. Chronic inflammation caused by long-term asbestos exposure is thought to be an important cause of MM, which is reported to occur in some organic mesothelial layers, such as the peritoneum, pleura, and pericardium, and even in the tunica vaginalis of the testis.

Although MM was described nearly a century ago, it is still difficult to diagnose in its early stages, and there is a lack of effective therapeutics due to our limited knowledge of its molecular pathogenesis. This has led to a generally poor prognosis for MM patients, with a 12-18 month median survival time [6–8]. The clinical manifestations of MM are usually nonspecific and insidious, resulting in a long incubation period of approximately 30-40 years, and diagnosis via advanced-stage computed tomography, positron emission tomography, and magnetic resonance imaging is not appropriate. Although both thoracoscopy and pathological examination are good ways to diagnose MM, it is invasive and inconvenient. Blood-based biomarkers are also regarded as an effective means for screening MM. Some traditional biomarkers of MM include soluble mesothelin, which is characterized by high specificity but low sensitivity [9]. In addition, fibulin-3 is useful for prognosis, and high values are statistically correlated with worse prognosis. Regardless, the value of fibulin-3 in MM diagnosis remains controversial [9–11]. Moreover, osteopontin levels may reflect inflammation, but the diagnostic value for MM is still under discussion [9, 12]. Recently, noncoding RNA-like microRNAs have been proposed as biomarkers for monitoring sensitivity to therapy and for prognostic purposes. Of course, the translation from lab research to clinical practice is often considered problematic [10]. Therefore, predictive early-stage or prognostic biomarkers that are clinically useful for MM require more active exploration.

Unfortunately, treatment options for advanced unresectable MM are very limited, and combination chemotherapy of cisplatin plus pemetrexed represents the most widely used regimen in the first-line setting for patients with unresectable MM [13]. More recently, immunotherapy has been suggested as a novel option for treating MM [14, 15]. For example, the programmed death-ligand 1 (PD-L1)/PD-1 pathway is an immunological checkpoint in cancer cells, and PD-L1 is expressed in malignant pleural mesothelioma (MPM) [16–18]. Anti-PD-L1/PD-1 inhibitors targeting the PD-L1/PD-1 pathway have been employed to treat patients with MPM, and efficacy is being investigated in several ongoing clinical trials [14, 19]. However, checkpoint blockade immunotherapy does have several limitations. For example, immune-related adverse events (irAEs) are unique side effects/toxicities that occur as a result of stimulating the immune system, and biomarkers predicting safety or predisposition toward irAEs are unfortunately lacking [14]. Similarly, methods for identifying patient populations that most benefit from checkpoint inhibition are scarce [14]. To improve prognosis, the recognition of this rare entity is as important as its early treatment. As there are serious unresolved public health issues regarding this asbestos-related cancer, novel and effective strategies for predicting the prognosis of, diagnosing, and treating MM are urgently needed.

In most mammalian cells, high-mobility group box 1 (HMGB1) acts as a nonhistone chromatin-binding protein that targets DNA and drives transcription factor assembly [20, 21]. Interestingly, nuclear HMGB1 also translocates to the cytosol and is then secreted into the extracellular environment [22, 23]. Extracellular HMGB1 actively secreted by innate immune cells, such as activated macrophages, neutrophils, and monocytes, functions as a proinflammatory cytokine, and it can also be released passively during cell injury or death [24, 25]. The acetylation status of HMGB1 is considered to play an essential role in the transfer process. Most nonacetylated HMGB1 is normally localized in the nucleus [26], whereas acetylation of the lysine residues in the two nuclear localization signals (NLS1/2) redirects HMGB1 to the cytoplasm [26, 27]. Notably, HMGB1 acetylation occurs in the nucleus and prevents HMGB1 from interacting with the nuclear-importer protein complex, thus blocking reentry of exported HMGB1 into the nucleus [28]. Interestingly, HMGB1 lacks a secretory signal peptide and does not traverse the endoplasmic reticulum-Golgi system; nonetheless, acetyl-HMGB1 can be packaged into secretory lysosomes and subsequently secreted into the extracellular space [28, 29]. In addition, the exocytosis and pyroptosis of HMGB1 are mediated by the inflammasomes in immune cells, also indirectly relying on acetylation [27, 30]. Inflammasome-induced HMGB1 release leads to extracellular, hyperacetylated HMGB1, which therefore can serve as a biomarker for pyroptotic cell death. In contrast, hyperacetylated HMGB is not generated during necrotic or apoptotic cell death [25, 30]. In addition to acetylation, methylation, phosphorylation, oxidation, glycosylation, and ADP-ribosylation of HMGB1 are involved in its cellular localization and immunological activity in the extracellular environment [31]. Furthermore, released HMGB1 can interact with cell surface receptors, such as the receptor for advanced glycation end products (RAGE), the toll-like family of receptors, and chemokine receptor-4, to induce their corresponding signaling pathways. These responses eventually lead to the activation of NF-*κ*B and the induction of activator protein 1 and mitogen-activated protein kinase pathways, which are strongly associated with triggering inflammation [32–34].

Notably, compared with normal healthy cells, a number of solid tumor cells exhibit increased expression and secretion of HMGB1, particularly in inflammation-associated cancers, such as hepatocellular carcinoma [35–37], colorectal cancer [38, 39], cervical carcinoma [40, 41], and MM [42–44], and high levels of HMGB1 are correlated with a poor clinical prognosis. In fact, the involvement of HMGB1 in cancer is complicated, and nuclear/intracellular and extracellular forms of HMGB1 have been demonstrated to participate in tumor development, progression, invasion, metastasis, and response to chemotherapeutics [32]. Because increasing evidence strongly suggests that HMGB1 is involved in MM and functions as a potential multiple biomarker or curative target, HMGB1 in MM has been a hot research topic in recent years.

## 2. Crucial Roles of HMGB1 in the Carcinogenic Mechanism in MM

**Figure 1:**
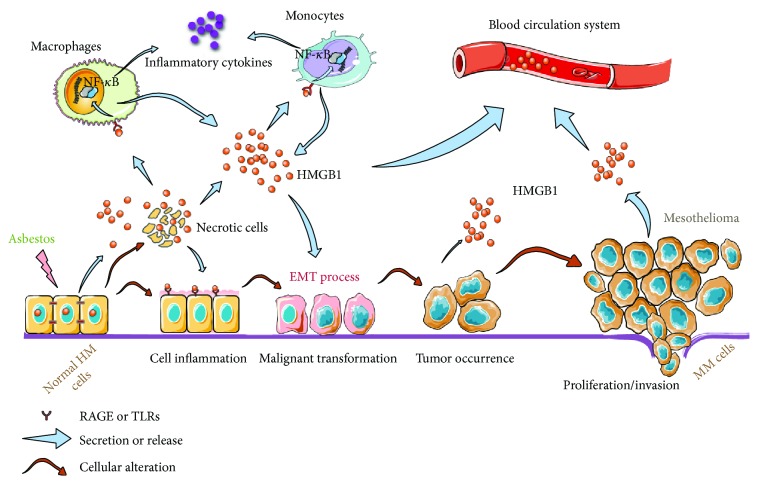
Schematic representation of HMGB1-involved mechanisms in asbestos-induced MM. See detail in text. HM cells: human mesothelial cells; MM: malignant mesothelioma; EMT: epithelial-to-mesenchymal transition; RAGE: the receptor for advanced glycation end products; TLRs: the Toll-like family of receptors.

Recently, a number of studies have supported the view that HMGB1 plays crucial roles in the tumorigenesis and development of MM. Here, we describe the three main roles of HMGB1 in the carcinogenic mechanism in asbestos-induced MM. Moreover, we propose possible HMGB1-involved mechanisms, as shown in Figure 1.

### 2.1. Asbestos-Induced Effector

Recent experimental evidence from cell lines and animal models of asbestos exposure suggests that HMGB1 is an asbestos-induced effector. Crocidolite and chrysotile are the two most typical mineral fibers used in biological experiments using models of asbestos exposure. For example, Yang et al. reported that crocidolite asbestos induced programmed necrosis and inflammation in the primary human mesothelial (HM) cells and that HMGB1 translocated from the nucleus to the cytoplasm and was released into the culture medium [45]. Furthermore, in crocidolite fiber-injected mice and hamsters, HMGB1 was observed in the nucleus, cytoplasm, and extracellular space of the mesothelial and inflammatory cells around asbestos deposits [45]. Moreover, sustained high serum levels of HMGB1 were caused by a high-dose, short-term injection protocol in the crocidolite fiber-injected mice. However, the increase in HMGB1 caused by chrysotile was transient, declining to background levels within 6 to 10 weeks. In addition, HMGB1 levels in mice injected with both crocidolite and chrysotile according to a low-dose, long-term protocol were higher than those in the vehicle negative control group for up to 10 weeks [46].

The above experimental evidence suggests that HMGB1 secretion by mesothelial or immune cells is highly responsive to asbestos fiber stimulation but may not be restricted to stimulation solely through exposure to asbestos fibers. Moreover, different types of asbestos fibers are likely to have different biological effects on endogenous HMGB1 secretion. Taken together, HMGB1 may act as an asbestos-induced effector.

### 2.2. Inflammatory Mediator

Chronic inflammation is a well-recognized tumor-enabling condition, and cellular inflammatory mediators in the tumor microenvironment are involved in almost all phases of cancer initiation, progression, and metastasis [47, 48]. Additionally, chronic inflammation is thought to be a hallmark of asbestos deposition in the tissue and to contribute to carcinogenesis [49]. In recent years, HMGB1 has been identified as a key mediator of inflammation- and damage-associated molecular patterns in a variety of inflammatory disorders and cancers [23, 32]. Asbestos fiber deposition induces a long-term inflammatory response. MM is considered a typical model of chronic inflammation-induced cancer associated with inhaled asbestos fibers. Once inhaled, asbestos fibers are likely rapidly attacked by macrophages and other immune cells and may disintegrate in the lungs into short fibers and particles and even form asbestos bodies [50]. Asbestos fibers also induce programmed necrosis and inflammation in the primary HM cells [45].

Subsequently, HMGB1 signals cellular damage in response to injury and inflammation. Upon exposure to asbestos, the proinflammatory cytokine HMGB1 prompts macrophages to secrete tumor necrosis factor-*α* (TNF-*α*), which protects mesothelial cells from asbestos-induced cell death and triggers a chronic inflammatory response [45]. Combined with previous findings, this evidence strongly suggests that HMGB1 may be a key inflammatory mediator involved in the carcinogenic mechanism underlying MM.

### 2.3. Cellular EMT Inducer

The epithelial-to-mesenchymal transition (EMT), a cellular process in which many molecular features of epithelial cells are lost and typical mesenchymal characteristics develop, including loss of cell-cell adhesion and cell polarity and acquisition of migratory and invasive abilities [51, 52]. Cancer cells that have undergone EMT are more aggressive and display increased invasiveness, stem-like features, and resistance to apoptosis [53]. EMT can also promote the production of proinflammatory factors by cancer cells [54]. Notably, increasing evidence emphasizes a link between cancer-associated EMT and chronic inflammation [51, 53, 54]. In fact, HMGB1 has been found to induce EMT in chronic inflammation-associated cancers, including colorectal carcinoma [55], intrahepatic cholangiocarcinoma [56], gastric cancer [57], cervical carcinoma [40], and MM [46, 58], and recent work supports the occurrence of HMGB1-induced EMT in HM cells exposed to asbestos, as well as in MM cancer cells. For instance, Qi et al. used microarray gene expression profiling to reveal that an epithelial marker of EMT, E-cadherin, was downregulated at the genetic level in the primary HM cells [46]. These *in vitro* results support the notion that EMT is activated in HM cells exposed to asbestos. Similarly, our group observed the characteristic cellular transformation of the EMT process in the normal HM line Met-5A cells subjected to long-term chrysotile asbestos exposure, with gradual appearance of the malignant phenotype. Furthermore, in the MM cell lines REN and Phi, HMGB1 significantly increased the levels of EMT signaling pathway components N-cadherin (a marker of mesenchymal differentiation) and *β*-catenin bound to the cytoplasmic tail of E-cadherin. Interestingly, cellular EMT signaling was repressed by the HMGB1 inhibitor salicylate [58].

These experimental results suggest that HMGB1 induces EMT in both asbestos-exposed HM cells and MM cancer cells and that it is associated with the malignant phenotype as well as the occurrence and migration of MM cells.

## 3. Possible Roles of HMGB1 as a Biomarker for MM

### 3.1. Predictive Biomarker of Asbestos Exposure

As mentioned in Section 2.1, HMGB1 may be released from necrotic or damaged mesothelial cells and activated inflammatory cells stimulated by asbestos fibers. In several populations from different countries, recent evidence has indicated that asbestos exposure can positively affect serum HMGB1 levels. One research group from the United States observed that HMGB1 levels in the serum of asbestos-exposed individuals (mean level: 80.2 ng/ml) were significantly higher than those of heavy smokers (mean level: 26.1 ng/ml) and nonexposed controls (mean level: 16.9 ng/ml) [45]. In another study, total HMGB1 serum levels were confirmed to be significantly higher in asbestos-exposed individuals (median level: 10.2 ng/ml) than in unexposed controls (median level: 1.4 ng/ml) [26]. However, the levels of hyperacetylated HMGB1 were very low in both healthy controls (median level: 0.5 ng/ml) and asbestos-exposed individuals (median level: 0.4 ng/ml), and there was no significant difference between the two groups. In addition, hyperacetylated HMGB1 comprised ~10% of the total HMGB1 in the serum of asbestos-exposed individuals [26].

Similarly, our research results in a Chinese Han population indicate that total serum HMGB1 levels in an asbestos-exposed (AE) group were significantly elevated relative to those in a nonexposed healthy group [59]. In detail, the median serum levels of HMGB1 in the two AE groups were similar at 50.06 ng/ml (AE < 10 years) and 50.42 ng/ml (AE ≥ 10 years), and both values were significantly higher than the median level of 41.68 ng/ml observed in healthy controls. However, no significant differences in HMGB1 levels were observed between the two groups with different exposure durations (AE < 10 and AE ≥ 10 years) AE [59]. These results suggest that one possible mechanism for asbestos-induced chronic inflammation is the secretion of HMGB1 into the stroma, after which it appears in the systemic circulation, regardless of asbestos exposure duration. According to the discrepancies between the HMGB1 median value in our results [59] and another report [26], it is suspected that HMGB1 levels do in fact vary according to ethnicity. Moreover, the storage times of blood samples and experimental conditions should also be considered as possible experimental reasons.

In summary, the serum level of HMGB1 is considered to be a predictive biomarker for monitoring occupational workers and their families who have a history of residential exposure to asbestos.

### 3.2. Blood-Based Diagnostic Biomarker

In view of the current difficulty in diagnosing MM, research on blood-based diagnostic techniques is of particular interest. Almost all of the literature to date supports that HMGB1 concentrations in the serum of patients with MM are significantly higher than those in the serum of healthy controls or AE individuals without MM [26, 43, 44, 59, 60]. Despite varying in different MM studies, the area under the curve (AUC) value is an effective index for evaluating diagnostic biomarkers. For example, the Japanese group Tabata et al. reported that serum HMGB1 levels of patients with MPM [43] or diffuse malignant peritoneal mesothelioma (DMPM) [60] were significantly higher than those of patients with benign asbestos-related diseases (ARD) and healthy AE individuals. AUCs of 0.674 (95% confidence interval (CI): 0.589-0.758) [43] and 0.821 (95% CI: 0.706-0.935) [60] were reported for MPM and DMPM patients, respectively. Furthermore, one research group from the United States showed that the total levels of HMGB1 exhibited high accuracy in discriminating MM patients from healthy controls, with an AUC of 0.999 (95% CI: 0.994-1.000) [26]. Moreover, when comparing AE individuals to healthy controls, the AUC of the total level of HMGB1 was 0.964 (95% CI: 0.893-1.000) [26]. Similarly, we found that in a Chinese Han population, HMGB1 levels in an MPM group were significantly higher than those of healthy control, AE and pleural plaque groups, with an AUC of 0.94 (95% CI: 0.89-1.03) for the ability of HMGB1 to distinguish MPM patients from healthy controls [59], though the AUCs for AE individuals with <10 years of exposure and ≥10 years of exposure were 0.81 (95% CI: 0.73-0.90) and 0.80 (95% CI: 0.72-0.89), respectively [59]. Moreover, Napolitano et al. reported that hyperacetylated HMGB1, a specific isoform, but not total HMGB1 reliably discriminated MM patients from asbestos-exposed individuals or healthy controls, with 100% specificity and sensitivity [26].

As described above, HMGB1 may serve as a potential biomarker for the clinical diagnosis of MM in high-risk AE cohorts. Of course, there are also limitations with using HMGB1 as a single index. For example, in one study, no significant differences between serum HMGB1 levels in patients with MPM and in those with lung cancer involving malignant pleural effusion were found [43]. Indeed, the very low AUC of 0.56 (95% CI: 0.39-0.73) observed when comparing MPM patients with AE individuals limits its clinical utility for identifying different types of ARD patients among large cohorts of AE or healthy individuals [59]. It is also worth mentioning that hyperacetylated HMGB1 may have better clinical diagnostic efficiency than total HMGB1 [26]. Overall, how acetylated HMGB1 differentiates MM from noncancerous conditions or from other cancers needs to be further explored. According to the current literature, hyperacetylated HMGB1 has only been detected in blood from patients with alcoholic liver disease [61], acute acetaminophen-induced liver failure [62], severe macrophage-activation syndrome [63], and drug-resistant epilepsy [64], and few epidemiological studies have described hyperacetylated HMGB1 levels in blood samples in patients with cancers other than MM. In addition, one challenge is that the only technique currently available for detecting HMGB1 isoforms is liquid chromatography-tandem mass spectrometry (LC-MS/MS). Thus, the development of accurate and rapid methods for clinical measurement that is less time-consuming and expensive than mass spectrometry is urgently required.

### 3.3. Pathologic Prognostic Biomarker

A number of studies have indicated that HMGB1 overexpression is associated with a worse prognosis in patients with several inflammation-associated cancers, such as hepatocellular carcinoma [35–37], colorectal cancer [38, 39], and cervical carcinoma [40, 41]. A similar result was also found in MPM, with a significant correlation revealed between serum HMGB1 levels and overall survival (OS) by Kaplan-Meier analysis [43]. In another recent study, an Italian research group analyzed the correlation between the HMGB1 immunohistochemistry scores for biopsy samples from MPM patients and disease-specific survival (DSS), and the HMGB1 score, especially total and cytoplasmic HMGB1 but not nuclear HMGB1, was negatively correlated with DSS [42]. In addition, because it is expressed in both normal and reactive mesothelial cells, the presence of HMGB1 in histologic MM samples is not suitable as a diagnostic biomarker [42].

Although the relationship between HMGB1 and OS or DSS still requires much more supporting evidence from a larger histologic sample number, HMGB1 can be considered a potential pathologic prognostic biomarker.

## 4. HMGB1-Targeting Therapeutic Strategies

Because of the crucial roles mentioned in Section 2, HMGB1 is becoming a potentially actionable target in molecular oncology for MM, with some recent preclinical evidence that suggests novel therapeutic approaches targeting HMGB1 in human MM, as listed in Table 1. These approaches interfere with asbestos-mediated inflammation, prevent or delay MM onset, and relieve the progression of MM. These findings also provide novel clues for the treatment of other chronic inflammation-induced cancers.

### 4.1. Recombinant HMG Box-A

Human HMGB1 consists of two DNA-binding domains (HMG Box-A and -B) and a 30-amino acid *C*-terminal tail. Interestingly, HMG Box-A, the truncated *N*-terminal domain, is known to be a specific antagonist of the full-length HMGB1 protein [65]. Conversely, HMG Box-B is capable of promoting cytokine secretion, similar to the proinflammatory activity of full-length HMGB1 [66]. Recombinant Box-A may compete with HMGB1 to bind to the HMGB1 receptor RAGE on the surface of activated macrophages but does not activate this receptor and instead shows significant anti-inflammatory activity [25, 65, 66]. In addition, recombinant Box-A antagonizes the cytokine activity of HMG Box-B [67], thereby displaying considerable anti-inflammatory activity.

Regarding MM-related studies, some experimental evidence has shown that recombinant HMG Box-A inhibits HMGB1 biological activity in cell and animal models. As an example, Yang et al. found that Box-A markedly reduces *in vitro* TNF-*α* secretion by macrophages treated with culture medium from AE HM cells [45]. It is noteworthy that TNF-*α* may activate NF-*κ*B, a signaling pathway that allows HM cells that have undergone asbestos-induced DNA damage to survive rather than die, thereby creating a pool of aneuploid mesothelial cells with the potential to develop cancerization [68]. Furthermore, Box-A suppresses the growth, migration, and viability of the REN mesothelioma cell line, though Box-A does not induce cytotoxicity in MM cell lines [44]. In an animal model, Box-A significantly reduced MM tumor growth in xenograft mice and extended the survival of mice injected with human MM cells, without side effects [58].

### 4.2. Anti-HMGB1 Neutralizing Monoclonal Antibody

Monoclonal antibodies (mAbs) have recently been extensively developed as molecularly targeted or immune-based intervention strategies. Anti-HMGB1 mAbs are promising in the development of novel therapies, and these neutralizing mAbs specifically bind to HMGB1 to inhibit its activity, with minimal side effects even at very high doses in experimental models. A number of studies have shown that anti-HMGB1 mAbs have potential therapeutic applications in many diseases involving HMGB1. For instance, in rat models of hemorrhage-induced brain injury and Parkinson's disease, anti-HMGB1 mAbs protected blood-brain barrier integrity and suppressed HMGB1 release from neurons and astrocytes into the extracellular space, reducing the level of HMGB1 in the blood as well as the expression of inflammatory cytokines [69, 70]. Interestingly, anti-HMGB1 mAbs had a long-term anti-seizure effect with minimal side effects in a mouse epilepsy model [71].

In MM studies, Jube et al. demonstrated that anti-HMGB1 neutralizing mAbs inhibited the activity of HMGB1 in REN cells and primary HM cells, blocked the HMGB1-RAGE interaction, and suppressed the MM malignant phenotype in severe combined immunodeficiency (SCID) mice with human MM xenografts [44]. These findings suggest that mAbs against HMGB1 inhibit its activity and the MM malignant phenotype by decreasing HMGB1 secretion and inflammatory factor expression.

### 4.3. Ethyl Pyruvate

Ethyl pyruvate (EP), a lipophilic derivative of pyruvic acid, is a safe and inexpensive compound with effective anti-inflammatory, antitumor, and cytoprotective activities [72]. EP may remove reactive oxygen species (ROS) from related cells in multiple inflammatory organ injuries [72, 73]. In particular, EP has the potential to inhibit tumor growth linked to inflammation [73], and many studies have investigated the effect of EP on tumors. EP inhibits hepatic [74–76], gastric [77], gallbladder [78], and prostate [79, 80] tumor growth *in vivo* and *in vitro* by inhibiting HMGB1 and downregulating the HMGB1-RAGE pathway.

Recently, EP has been confirmed to act as an effective inhibitor of HMGB1 [65]. A research group from the USA investigated the effect of HMGB1 targeting by EP on the suppression of the malignant phenotype of human mesothelioma [81]. These authors reported that EP effectively inhibited HMGB1 localization and secretion in MM cell lines REN and HP3 (also referred to as Phi), and they utilized REN and HP3 cells to demonstrate that EP suppressed the viability, motility, migration, and anchorage-independent growth of MM cells by hampering HMGB1 release mediated through inhibition of NF-*κ*B nuclear translocation [81]. REN cells are derived from an explant of a patient with an epithelial mesothelioma [58], and HP3 cells were isolated from the effusion of an epithelial mesothelioma patient [82]. Epithelioid is the most common subtype of MM [83], though there might be a limitation for the other two major subtypes, sarcomatoid and biphasic, of MM. Moreover, EP treatment decreased the serum levels of HMGB1 in the SCID mice with human MM xenografts.

### 4.4. Aspirin and Its Metabolite Salicylic Acid

Aspirin, also known as acetylsalicylic acid (ASA), is a type of nonsteroidal drug with a well-characterized anti-inflammatory effect [84]. Aspirin and its metabolite salicylic acid (SA) are widely used to treat fever and inflammation-mediated diseases and to prevent cardiovascular disease [85]. After being absorbed by the gastrointestinal tract, aspirin is quickly hydrolyzed to SA in the gastrointestinal tract, liver, and blood. Much of the bioactivity of ASA is attributed to SA because the latter is effective in the blood for several hours [85].

As discussed in Section 2.2, inflammation induced by HMGB1 contributes to malignant mesothelioma. SA suppresses the proinflammatory activities of HMGB1 by directly binding to it [86], and data show that aspirin and SA reduce the level of extracellular HMGB1 secreted by human MM cells [58]. Furthermore, the serum level of HMGB1 was reduced in the SCID mice injected with human MM cells treated with ASA [58]. MM cell growth, motility, migration, and invasion, as well as EMT signaling, play critical roles in the HMGB1-dependent tumorigenesis and progression of mesothelioma, and ASA inhibits HMGB1, thereby suppressing mesothelioma growth [58].

### 4.5. Flaxseed Lignans

Flaxseed, i.e., the seed of the flax plant, has been a part of the human diet worldwide for thousands of years [87]. One of the main components of flaxseed is lignans, the concentrations of which are higher in flaxseed than in other plants, and 95% are composed of secoisolariciresinol diglucoside, a lignan precursor [88]. The lignans found in plants are phytoestrogens that are structurally similar to endogenous estrogens but can have both estrogenic and antiestrogenic effects [89].

One research group from the United States showed that the flaxseed lignan component (FLC) could prevent acute asbestos-induced inflammation in a mouse model [90]. The HMGB1 concentration in the peritoneal lavage fluid (PLF) of FLC-fed MM-prone Nf2^+/mu^ mice was significantly decreased relative to that in the control group, which corresponded to a decrease in HMGB1 mRNA levels in the total white blood cells of the PLF [90].

## 5. Conclusion and Future Perspectives

As we describe herein, findings to date provide new insight into the molecular mechanisms underlying the progression and prognosis of MM and may lead to new approaches for the effective diagnosis and therapy of MM.

It is essential to investigate the multifaceted role played by HMGB1 in MM. First, the possible functions of HMGB1 in asbestos-induced MM have recently attracted particular concern in occupational tumor research. The underlying mechanisms by which HMGB1 is involved in MM cell growth, motility, migration, and invasion remain to be elucidated. Second, HMGB1 is a useful serum biomarker for screening MM patients, and it has diagnostic and prognostic value for evaluating high-risk AE cohorts or the prognosis of MM. Nevertheless, there are many inflammation-associated diseases or extrinsic factors that cause elevated levels of HMGB1. Hence, the combination of HMGB1 levels, other biomarkers, and radiographic findings may be helpful for identifying MM in AE populations. Translational modifications of HMGB1 might involve serving as a marker for the type of cell death that has occurred. Third, a number of studies have suggested that HMGB1 is a potential therapeutic target in ARDs, and novel strategies targeting HMGB1 should be further developed and may be beneficial in medical treatments for related diseases. In the future, new strategies will provide specific therapies for interfering with asbestos-induced inflammation to prevent or delay the onset and relieve the progression of MM.

## Figures and Tables

**Table 1:** Novel strategies targeting HMGB1 in malignant mesothelioma.

Types	Substances	Biological effects on HMGB1	Cell models	Animal models	References
Polypeptides	Recombinant HMG Box-A	Recombinant HMG Box-A inhibits HMGB1 activity with more efficient HMGB1 targeting.	Phi cells	MM xenograft mouse model (injected with the human MM cell line REN)	[58]
			Primary HM cells	—	[45]
			REN cells, PPM-Mill cells, PPM-Phi cells	—	[44]
	Anti-HMGB1 neutralizing monoclonal antibody	An anti-HMGB1 neutralizing monoclonal antibody inhibits HMGB1 and the MM malignant phenotype.	REN cells, primary HM cells	SCID mice with human MM xenografts	[44]
Chemical pharmaceuticals	EP	EP affects the localization and secretion of HMGB1 in MM cells. EP decreases serum HMGB1 levels in MM xenografts.	REN cells, HP3 cells	Orthotopic MM xenograft mouse model	[81]
	Aspirin and its metabolite, salicylic acid	Aspirin and its metabolite salicylic acid reduce the serum level of HMGB1 and suppress the secretion of HMGB1 by MM cells.	REN, HMESO, PPM-MILL, and Phi cells (primary MM cells)	Xenograft SCID mouse model (injected with the human MM cell line REN)	[58]
Plant extracts	Flaxseed lignans	Flaxseed lignans reduce HMGB1 gene expression and secretion in the blood.	—	MM-prone Nf2^+/mu^ mouse model	[90]

MM: malignant mesothelioma; HMG: high-mobility group; HMGB1: high-mobility group box 1 protein; HM cells: human mesothelial cells; SCID: severe combined immunodeficiency; BBIs: bromodomain inhibitors; PARP: poly (ADP-ribose) polymerase; EP: ethyl pyruvate.
